# Research progress in unilateral moyamoya disease

**DOI:** 10.3389/fnhum.2025.1503639

**Published:** 2025-01-27

**Authors:** Xiaokuan Hao, Cunxin Tan, Ziqi Liu, Yang Tie, Yanru Wang, Shihao He, Ran Duan, Rong Wang

**Affiliations:** ^1^Department of Neurosurgery, Beijing Tiantan Hospital, Capital Medical University, Beijing, China; ^2^Department of Neurosurgery, Peking University International Hospital, Beijing, China; ^3^Department of Neurosurgery, Peking Union Medical College Hospital, Peking Union Medical College and Chinese Academy of Medical Sciences, Beijing, China; ^4^China National Clinical Research Center for Neurological Diseases, Beijing, China

**Keywords:** unilateral moyamoya disease, etiology, epidemiology, clinical course, progression, surgery

## Abstract

Unilateral moyamoya disease (U-MMD) is a chronic vascular disease characterized by progressive stenosis and occlusion of the terminal end of the internal carotid artery and its main branches, resulting in the appearance of moyamoya-like blood vessels at the base of the brain. The etiology of U-MMD is unknown, it accounts for 9.7–17.8% of all moyamoya disease, and the family incidence is 5.5–13.3%. The clinical characteristics are similar to those of typical moyamoya disease, but there are some differences. U-MMD can progress to bilateral moyamoya disease with a median probability of 29.01% (ranging from 6.3 to 58.8%), and there are many risk factors that promote its development. Surgical treatment can effectively reduce the incidence of ischemic stroke and improve prognosis. However, the timing and indications for surgery require further investigation. This article reviews the latest research progress on the etiology, epidemiology, clinical and radiological characteristics, progression, treatment, and prognosis of U-MMD.

## Introduction and background

**Figure 1:**
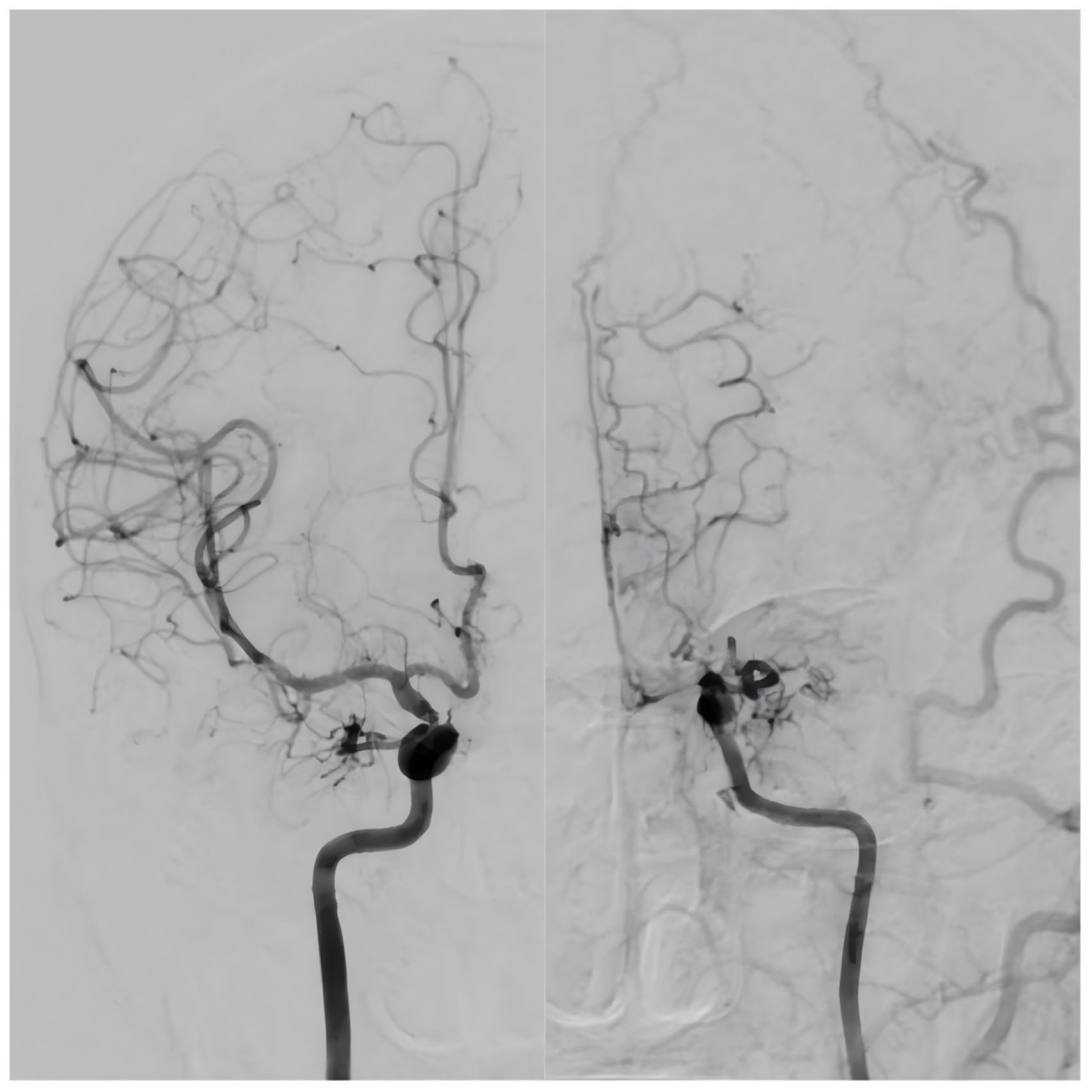
Typical unilateral moyamoya disease.

Moyamoya disease (MMD) is a chronic cerebrovascular occlusive disease characterized by the development of stenosis or occlusion at the terminal portion of the internal carotid artery (ICA) and/or initial part of the anterior cerebral artery (ACA) and middle cerebral artery (MCA), accompanied by the formation of abnormally intensive moyamoya-like vessels (Suzuki and Takaku, 1969). In 2012, the Japanese guidelines for the diagnosis and treatment of MMD (On the Pathology, Research Committee, 2012) introduced the concept of unilateral MMD (U-MMD), in which one side exhibits typical MMD-like changes and the opposite side exhibits normal or mild narrowing of the blood vessels (Figure 1). Since 2021, the new guidelines (Kuroda et al., 2022) include U-MMD as a possible manifestation of MMD. U-MMD can progress to typical MMD (Baba et al., 2008), there is a controversy about its development process. Some scholars believe that U-MMD is an independent subtype of MMD (Houkin et al., 1996; Seol et al., 2006), while others believe that U-MMD is an early manifestation of typical MMD (Tian et al., 2022; Kim et al., 2012). This review focuses on recent novel research regarding the etiology, epidemiology, clinical characteristics, radiological characteristics, progression, treatment, and prognosis of U-MMD. The relevant literature over the past 20 years is presented in Table 1.

**Table 1:** Relevant literature published in the past 20 years.

Study	Ethnic origin	Cases	Patients	Duration	Results
Kelly et al. (2006)	American	18	Children and adults	17.1 ± 3.5 months	The presence of minor changes in the contralateral ACA, intracranial ICA and MCA is a risk factor
Smith and Scott (2008)	American	33	Children and adults	5.3 years	The progression rate was 22.2%, the mean time to progression was 2.2 years, they reported a number of risk factors
Park et al. (2011)	Korean	34	Children only	35.3 months (18.1–100)	The progression rate was 58.8%. PCA involvement is a risk factor.
Yeon et al. (2011)	Korean	45	Children only	53.4 months (13–157)	Age < 9 years old was the only independent predictor of contralateral progression
Chen et al. (2015)	Chinese	68	Children and adults	NA	The overall autoimmune disease prevalence in the U-MMD was significantly higher
Zhang et al. (2016)	Chinese	109	Children and adults	43.8 ± 21.3 months	A one-peak pattern in age distribution, lower grade of Suzuki stage, fewer familial cases, and PCA involvement were observed in patients with U-MMD
Lee et al. (2020)	Korean	146	Children and adults	4.3 ± 2.4 years	Endothelial shear stress is a risk factor
Church et al. (2020)	American	217	Children and adults	5.8 years (1–22)	The progression rate was 8.3%. Baseline stenosis and hyperlipidemia are risk factors.
Ok et al. (2022)	Korean	123	Children and adults	18–54 months	RNF213 p.R4810K mutation is a risk factor
Zhang et al. (2022)	Chinese	45	Adults only	4–12 months	Surgery can significantly improve cerebral blood flow
Tian et al. (2022)	Chinese	89	Adults only	63 ± 15 mouths	The progression rate was 22.2%.
Strunk et al. (2023)	Caucasian	63	Children and adults	5.4 ± 3.7 years	The progression rate was 22.2%. PCA involvement is a risk factor.
Mineharu et al. (2024)	Japanese	93	Children and adults	NA	In U-MMD, there is a gene dose effect of the p.R4810K mutation on PCA involvement
Suo et al. (2024)	Chinese	a family with U-MMD	Children and adult	NA	FOXM1 c.1205C > A mutation potentially contributes to U-MMD pathogenesis.
Wang et al. (2024a)	Chinese	83	Children and adults	7.9 ± 2.0 years	RNF213 p.R4810K mutation, younger age, and PCA involvement are risk factors. Obvious surgical effect

## Epidemiology and clinical characteristics

In recent years, a large number of studies have been conducted on the epidemiology of U-MMD, and the number of cases has increased. However, there is some variation in research data between different countries and ethnic groups.

Kelly et al. (2006) reviewed MMD patients at the Stanford University Medical Center, among whom U-MMD accounted for 17.8%, which is the highest proportion among all studies; however, their study population included almost all races except Black individuals. Yeon et al. (2011) reported that U-MMD accounted for 11.4% of the total number of children and adolescents with MMD in the Korean population. Kim et al. (2012) showed that adults with U-MMD accounted for 14.9% of the total number of Japanese population. Duan et al. (2012) conducted a single-center study with the largest number of cases to date (802), showing that U-MMD accounts for 9.7% of the total number of patients with MMD in the Chinese population. Hayashi et al. (2013) conducted an epidemiological study on MMD across Japan and showed that U-MMD accounted for 10.5% of the total number of patients with MMD in the Japanese population. All of the above studies reported prevalence rates; as can be seen, the proportion of U-MMD in patients with MMD is not high, at approximately 15% on average.

Most studies have reported that the number of female patients with U-MMD is higher than that of male patients, and the female-to-male ratio is approximately 1.5:1 (Ikezaki et al., 1997; Hayashi et al., 2010; Chen et al., 2016), which is consistent with the sex ratio of typical MMD (Scott and Smith, 2009). Some studies have shown no sex differences in U-MMD (Zhang et al., 2016; Smith and Scott, 2008; Wang et al., 2024a; Strunk et al., 2023).

Most studies have shown that the age distribution of patients with U-MMD is bimodal; one group is children around 10 years old, and the other is adults around 40 years old, which is not significantly different from the age distribution of typical MMD (Kelly et al., 2006; Hayashi et al., 2014; Ogata et al., 2008). Some research results show that the other peak of onset is at approximately 50 years of age, which is slightly different (Duan et al., 2012; Wang et al., 2024a). However, a large retrospective study by Zhang et al. (2016) in China showed that age distribution was unimodal, ranging from 30 to 39 years.

Most studies have reported that patients with U-MMD have a family history of the disease. Ikezaki et al. (1997) retrospectively analyzed 180 patients with U-MMD and reported a family incidence rate of 6.7%. Zhang et al. (2016) reported the familial occurrence was 5.5%, whereas in 2024, Wang et al. (2024a) reported it was 13.3%. Most scholars believe that the initial symptoms of U-MMD are similar to those of bilateral MMD, particularly cerebral ischemia (Zhang et al., 2016; Hirotsune et al., 1997; Acker et al., 2015). Transient ischemic attack (39.4%) was the most common symptom at onset (Hayashi et al., 2014). Interestingly, U-MMD demonstrates different characteristics in terms of bleeding patterns compared to bilateral MMD. Yu et al. (2019) reported that subarachnoid hemorrhage was more common in patients with U-MMD whereas intraventricular hemorrhage was more common in patients with bilateral MMD, and the Suzuki staging of acute intracranial hemorrhage was lower in patients with U-MMD than in patients with bilateral MMD. Additionally, the incidence of cerebral hemorrhage was higher in adults with U-MMD (32.8%) than in those with bilateral MMD (27.0%) (Ikezaki et al., 1997).

## Etiology

The etiology of U-MMD remains unknown. Previous studies have focused on genetic factors, immune inflammation, and susceptibility genes in U-MMD (Baba et al., 2008; Zhang et al., 2016). With regard to genetics, the question of whether U-MMD is hereditary and whether it represents an early manifestation of MMD or a distinct pathological entity has been controversial. Houkin et al. (1996) reported that U-MMD was not inherited by studying 10 cases of pediatric U-MMD. However, in recent years, an increasing number of studies have shown that it has a certain familial inheritance. Kusaka et al. (2006) reported a case of U-MMD with typical MMD occurring in the same family, suggesting that they are different phenotypes of the same gene. In the same year, Mineharu et al. (2006) conducted chromosomal analyses of 6 patients with a family history of U-MMD and demonstrated that familial U-MMD was inherited in an incomplete autosomal dominant pattern. The above study showed that the heritability of U-MMD was 5.5–10%, which was lower than the 15% heritability of typical bilateral MMD (Scott and Smith, 2009), suggesting that there may be hitherto unknown inheritance patterns. In the area of metabolism and immunology, Houkin et al. (1996) studied basic fibroblast growth factor (bFGF) in cerebrospinal fluid of 10 children with U-MMD, and found that a decrease in bFGF was closely related to U-MMD. However, Jeon et al. (2015) performed a metabolomic analysis of cerebrospinal fluid in adults with U-MMD using a larger sample size; however, the results showed that there was no difference in cerebrospinal fluid metabolite levels between patients with U-MMD and those with bilateral MMD. Hence, future studies on cerebrospinal fluid metabolites of U-MMD are warranted.

Immune disorders may also be a cause of U-MMD. Chen et al. (2015) followed up 25 MMD patients with Graves’ disease and found more frequent progression to U-MMD during the 106.4 ± 48.6 month follow-up period. Soon after, Chen et al. (2016) retrospectively analyzed the clinical characteristics of 316 patients with bilateral MMD and 68 patients with U-MMD and found that the overall prevalence of autoimmune diseases in patients with U-MMD was higher than that in patients with bilateral MMD. Because U-MMD shows a higher association with autoimmune diseases than bilateral MMD, different pathogenic mechanisms may be involved in the formation of diseased blood vessels. To date, no mechanistic studies have examined how immune factors contribute to the pathological angiogenesis that occurs in U-MMD; however, advances in gene sequencing technology has allowed the identification many susceptibility genes, which has helped to advance the study of the disease mechanisms. The *RNF213* (ring finger protein 213) gene is currently the most studied susceptibility gene in U-MMD. Ok et al. (2022) performed sequencing analysis on 123 patients with U-MMD and detected an *RNF213* p.R4810K gene mutation in 72 patients, all of which were heterozygous. The allele frequency of *RNF213* p.R4810K was significantly higher in the U-MMD group than in the control group. Mineharu et al. (2024) analyzed a cohort of 93 patients with U-MMD and found that posterior cerebral artery (PCA) involvement in U-MMD was exclusively ipsilateral and was influenced by the *RNF213* mutation gene dosage. In the same year, Suo et al. (2024) identified candidate genes by performing whole-genome sequencing of a family with U-MMD, and their results suggested that the *FOXM1* c.1205 C > A mutation potentially contributes to U-MMD pathogenesis. In the most recent study in 2024, Wang et al. (2024a) followed up 83 cases of U-MMD for 7.9 ± 2.0 years and found that heterozygous *RNF213* p.R4810K mutations occurred significantly more frequently in U-MMD patients with contralateral progression. In conclusion, these susceptibility genes have an impact both in the pathogenesis and progression of U-MMD, and future studies will focus on the downstream pathways and cellular function of the genes.

## Progression

It is important to study the progression of U-MMD by identifying the risk factors for progression so that personalized follow-up and treatment can be provided before a stroke occurs. So far, there have been 18 studies on the development of U-MMD into bilateral MMD [1 from the USA, 1 from Germany, 3 from China, 6 from Japan, and 7 from South Korea (Houkin et al., 1996; Seol et al., 2006; Tian et al., 2022; Kim et al., 2012; Kelly et al., 2006; Yeon et al., 2011; Hayashi et al., 2010; Zhang et al., 2016; Wang et al., 2024a; Strunk et al., 2023; Hirotsune et al., 1997; Ok et al., 2022; Kawano et al., 1994; Kuroda et al., 2005; Joo et al., 2011; Matsushima et al., 1994; Park et al., 2011; Lee et al., 2020)], all of which focused on vascular progression evident on imaging rather than progression in clinical symptoms. Three studies focused only on adult patients, six studies focused solely on pediatric patients, and the rest were not age-specific. However, only 4 studies on U-MMD had a sample size >100. The median rate of progression in each study was 29.01% (range, 6.3–58.8%), and the average progression time varied from 1 to 78 months (Tian et al., 2022). Some scholars (Kim et al., 2012; Duan et al., 2012) believe that among the risk factors for predicting progression, patients with young age, slight stenosis of the contralateral vessels, and familial factors are more likely to have contralateral progression. Smith and Scott (2008) reported that contralateral vascular abnormalities, congenital heart malformations, previous brain irradiation, Asian lineage, and familial MMD syndrome in patients with U-MMD increased the risk of disease progression.

Previous studies have shown that U-MMD in pediatric patients is more likely to progress to the contralateral side than U-MMD in adult patients. Smith and Scott (2008) retrospectively analyzed 33 patients with U-MMD, including 4 adults and 29 children. The results showed that children younger than 7 years were prone to rapid progression, with an average time of 10.8 months, whereas patients older than 7 years showed slow progression, with an average of 37.2 months. Park et al. (2011) studied 34 pediatric patients and concluded that U-MMD progression was related to age. Patients younger than 8 years easily experienced rapid progression, with an average time of 14.18 months. Patients older than 8 years showed slower progression, with an average time of 22.38 months. The rates of progression in the two studies described above were 30 and 58.8% respectively; these ratios seem to be quite high, which may be because the study included children with mild contralateral vascular stenosis, thus creating mixed factors in the target population. Yeon et al. (2011) enrolled 45 patients with typical U-MMD from a cohort of 391 children using strict inclusion criteria and excluded children with mild contralateral vascular stenosis. The results showed that the progression rate was only 17.8% during the 53.4 month follow-up period. They reported that among the 45 children with U-MMD, those younger than 9 years were prone to progression, while those older than 9 years had slower progression. Compared with Smith, the results showed that there was no significant difference in the incidence of contralateral progression between patients younger than 7 years and those younger than 9 years, and contralateral progression is likely to occur within 1.8 to 3.1 years after initial diagnosis, regardless of age.

As the understanding of U-MMD continues to grow, and the sample sizes of studies increase, the evidence becomes more compelling. The first large study was a retrospective review of 109 cases by Zhang et al. (2016), which excluded children with mild contralateral ACA, MCA, or ICA stenoses. The results showed that the incidence of contralateral progression in all cases was only 16.5% over a mean follow-up of 43.8 ± 21.3 months, which was lower than previous studies. To date, the largest sample size was a study of 217 cases of U-MMD performed by Church et al. (2020), with an average follow-up time of 5.8 years (range 1–22 years). The results showed that only 8% of patients experienced contralateral progression. Tian et al. (2022) followed 89 adults with U-MMD for 63 months and found that only 9% experienced contralateral progression. Their study also excluded patients with mild contralateral ACA, MCA, or ICA stenoses. With the improvement of scientific inclusion criteria, it has been proven that the progression of U-MMD in 5 years is not as high as previously reported. It is worth mentioning that in 2024, Wang et al. reported 19/83 (22.9%) of patients with U-MMD in the study experienced contralateral progression during a mean follow-up duration of 7.9 ± 2.0 years (range 2.0–13.9 years). This result is also reasonable because the follow-up period is the longest thus far, and the progression rate is expected to increase over time.

Several studies have reported various risk factors for contralateral progression. Younger age was confirmed as a predictive factor for contralateral progression. From the above discussion, we can see that for pediatric U-MMD patients aged less than 9 years, it is necessary to conduct more frequent clinical follow-up and timely disease management. Contralateral stenosis of the ACA, MCA, or ICA has been identified as a risk factor for contralateral progression (Zhang et al., 2016; Smith and Scott, 2008), whereas Yeon et al. (2011) reported no association between stenosis of the contralateral A1 segment and contralateral progression.

Mineharu et al. (2024) reported that PCA involvement in U-MMD is exclusively ipsilateral and influenced by *RNF213* mutation gene dosage. Wang et al. (2024a) reported that *RNF213* p.R4810K mutations and PCA involvement were predictors of contralateral progression. Previous studies have reported that patients with the *RNF213* p.R4810K mutation (Wang et al., 2020) or PCA involvement (Mugikura et al., 2002; Hishikawa et al., 2013) in classic MMD exhibit more severe disease and more extensive vascular involvement, which can reasonably explain the results of Mineharu and Wang’s study on susceptibility to contralateral progression. Strunk et al. (2023) also reported that PCA involvement and dizziness are significantly more frequent in patients with progressive MMA. In addition, Church et al. (2020) found that hyperlipidemia was a predictor of contralateral progression, and Strunk et al. (2023) and Mineharu et al. (2024) confirmed this phenomenon; they believed that elevated lipids have a deleterious effect on the underlying moyamoya vasculopathy.

Few studies have examined the mechanisms of how U-MMD develops; only Lee et al. (2020) reported a mechanism by which endothelial shear stress causes contralateral progression of U-MMD. Endothelial shear stress parameters were mean and maximum signal intensity gradients at the vessel boundary in time-of-flight sequences from brain magnetic resonance imaging; they believed that the sharp bends and accelerated flow rate at the terminal end of the ICA lead to high spatial variability of endothelial shear stress. Subsequently, this upregulates transcription factors and modulates endothelial gene expression.

## Radiologic characteristics

Ogata et al. (2008) studied the pattern of collateral circulation in patients with U-MMD and found that there was no collateral circulation via the ocular artery in patients with U-MMD, but there was increased collateral circulation from the contralateral normal vessels. Other sources of collateral circulation were not significantly different from those in patients with typical MMD. In 2014, a retrospective study of 203 U-MMD cases in Japan showed that U-MMD patients had lower Suzuki stages (Hayashi et al., 2014). In 2016, a study of 109 patients with U-MMD by Acker et al. (2015) showed that 70% of patients with U-MMD had a lower Suzuki stage, which was significantly higher than that of patients with typical MMD (50%). PCA involvement occurs in approximately 24.5–48.6% of patients with typical MMD and is often bilateral (Wang et al., 2020; Miyatake et al., 2012). However, in 2024, a study of 93 cases of U-MMD by Mineharu et al. (2024) reported that only 11.8% of patients with U-MMD manifested PCA involvement, which was significantly lower than that in patients with typical MMD, and that PCA involvement in U-MMD was exclusively ipsilateral.

Although only one hemisphere is involved in U-MMD, there is also a decrease in regional cerebral blood flow (rCBF). Patients with U-MMD have unique perfusion characteristics, as evidenced by reduced local rCBF in the frontotemporal lobe and elevated rCBF in the occipital lobe. The rCBF of U-MMD patients after acetazolamide stimulation was significantly higher than that of typical MMD patients, and their ability to respond to acetazolamide was also higher (Ogawa et al., 1987). A previous study showed that smoky vessels gradually developed with a decrease in rCBF, which also explains the lower Suzuki grade of patients with U-MMD (Ogawa et al., 1990). These results are consistent with those of previous studies (Mugikura et al., 2002), showing that the frequency of PCA involvement is higher in the hemisphere with an advanced Suzuki stage. These observations seem to support a previous hypothesis that MMD belongs to a clinical entity of neurocristopathy, which suggest that neural crest cell migration predominantly occurs ipsilaterally (Tucker and Lumsden, 2004).

## Treatment and prognosis

Surgery can improve cerebral perfusion, reduce the incidence of cerebral ischemia, and improve the prognosis of U-MMD (Fung et al., 2005). Zhang et al. (2016) reported that among 109 patients with U-MMD, most had a good functional prognosis after surgery, and approximately 91.7% had a modified Rankin Scale score of 0–2 during follow-up, while the percentage of overall MMD patients who could achieve this score was 84.6%. Zhang et al. (2022) studied the hemodynamic characteristics of 45 patients with U-MMD after bypass grafting, and the results showed that the cerebral blood flow, cerebral blood volume, mean transit time, and time to peak were significantly improved; furthermore, the neurological function of all patients was good. Wang et al. (2024a) reported that of 19/83 (22.9%) patients with U-MMD achieved significant neurological improvements at Matsushima stages A and B after surgery. In conclusion, for U-MMD, surgical plans should be actively evaluated to reduce the risk of stroke. In addition, given its unique PCA involvement pattern, increased attention should be given to follow-up of PCA involvement development (Bao et al., 2023), and ipsilateral posterior circulation revascularization should also be considered if necessary (Wang et al., 2024).

Whether the contralateral normal hemisphere in patients with U-MMD requires surgery remains unclear. An early study by Matsushima et al. (1988) recommended that patients with U-MMD younger than 2 years should undergo bilateral reconstruction because the incidence of normal-side progression is high. The asymptomatic side in patients with U-MMD should be treated surgically, particularly in children. However, some studies (Nagata et al., 2006) have indicated that surgical treatment should be postponed until symptoms appear on the asymptomatic side. As discussed previously, an in-depth study of the natural history of U-MMD has shown that the probability of contralateral progression is low; therefore, an increasing number of scholars believe that prophylactic contralateral revascularization is not warranted in U-MMD cases (Tian et al., 2022; Zhang et al., 2016). Instead, careful and long-term follow-up is more conducive for the early detection of contralateral progression, thereby determining the surgical strategy in a timely manner, especially for patients with risk factors. It is worth mentioning that U-MMD should be strictly distinguished from atherosclerotic disease in future studies (Psychogios et al., 2022), as they differ in clinical features and surgical prognosis (Wang et al., 2024b).

## Prospects and challenges

With the increase in U-MMD research and the development of imaging technology, the incidence of U-MMD has increased annually, so it is receiving increasing attention. However, the studies are still not sufficiently thorough, and many problems have not been solved. First, does U-MMD demonstrate a unique pattern in terms of epidemiology, such as clinical symptoms and heritability, compared to typical bilateral MMD? What factors contribute to this development? The reason for the variable results across studies is that there are no prospective cohort studies on the natural course of U-MMD and only a few large retrospective studies. Therefore, the next step is to conduct large multicenter prospective clinical trials. In addition, the high number of risk factors for U-MMD progression to bilateral MMD and the lack of evidence for their use in clinical diagnosis and treatment requires more evidence-based medical studies to support the indications and methods of surgical treatment. Finally, there remains a gap in the study of U-MMD vascular progression at the cellular and organ levels, and further basic proteomic and organ-focused studies are urgently required.
